# Pre-operative physiotherapy for elderly patients undergoing abdominal surgery

**DOI:** 10.4102/sajp.v78i1.1782

**Published:** 2022-09-27

**Authors:** Rozelle Labuschagne, Ronel Roos

**Affiliations:** 1 Department of Physiotherapy, Faculty of Health Sciences, University of the Witwatersrand, Johannesburg, South Africa

**Keywords:** abdominal surgery, DEMMI, elderly, function, pre-operative, prehabilitation, physiotherapy, 6-minute walk test

## Abstract

**Background:**

Elderly patients report a decrease in function and activities of daily living following abdominal surgery. The objectives of our pilot study were to determine the effects of a single pre-operative physiotherapy session consisting of education and exercise on clinical and physical function outcomes in elderly patients.

**Methods/design:**

A single-blind pilot randomised controlled trial evaluated clinical and functional outcomes of elderly patients following surgery in a private hospital in Pretoria, South Africa. The outcomes included length of hospital stay (LOS), postoperative pulmonary complications (PPC), first mobilisation uptime, DeMorton Mobility Index (DEMMI), 6-minute walk test (6MWT), Lawton–Brody’s instrumental activities of daily living (IADL) and the Functional Comorbidity Index (FCI). Descriptive and inferential statistics were undertaken, and statistical significance was set at *p* ≤ 0.05.

**Discussion:**

Twelve participants (*n* = 11 female [91.67%] and *n* = 1 [8.33%] male) with a mean age of 65.75 (±4.47) years were included. Most participants (*n* = 10, 83.33%) underwent lower abdominal laparotomy (*n* = 10, 83.33%). The median hospital LOS was *n* = 4 (IQR 3.25–4) days; walking distance at first mobilisation was 130 m (IQR (85–225), with intervention participants walking further (intervention: 177 m, IQR 100–242.50; control: 90, IQR 60 m – 245 m; *p* = 0.59). Recruitment was low, with only 10.95% referrals and 47.82% nonconsents.

**Conclusion:**

A single physiotherapy session prior to surgery demonstrated a potential favourable change in elderly patients’ mobility postoperatively; however, further research is necessary.

**Clinical implication:**

A once-off pre-operative physiotherapy session could enhance recovery in elderly patients.

**Trial registration:**

Pan African Clinical Trial Registry, PACTR201809874713904, https://pactr.samrc.ac.za/TrialDisplay.aspx?TrialID=3593

## Introduction

Surgical procedures have become a frequent medical intervention as disease patterns, treatments and technology change, resulting in a worldwide rise in procedures (Weiser et al. 2016). In 2012, an estimated 4991 operations per 100 000 people were performed in South Africa, accounting for nearly 3 million operations per year (Weiser et al. 2016). Abdominal procedures account for a large portion of these surgeries (Reeve & Boden 2016). Following abdominal surgery, up to 35% of patients may experience surgical or medical complications (Pouwels et al. 2014; Reeve & Boden 2016). Surgical complications could include wound infections, abscess formation, bleeding, hernias, ileus and septicaemia (McGillicuddy et al. 2009; Tahiri et al. 2016; Whelan et al. 2021). Medical complications may include pulmonary, cardiovascular and urinary complications such as pneumonia, atelectasis, deep vein thrombosis, arrhythmia, urinary tract infections, renal failure and delirium (McGillicuddy et al. 2009; Schiphorst et al. 2015; Tahiri et al. 2016). The elderly are at high risk of developing postoperative pulmonary complications (PPC), partly because of the respiratory system changes that occur with aging (Lalley 2013).

Changes with age are aggravated with disease and inactivity but can be modified with exercise (Navaratnarajah & Jackson 2013). Structural and physiological changes to the thoracic cage, lungs and diaphragm affect chest wall and lung compliance as well as respiratory muscle strength (Dyer 2012; Navaratnarajah & Jackson 2013). Stiffness of the thoracic cage and chest wall leads to weakened inspiratory and expiratory muscles in the unused ranges, which then compounds the effect of age on effective lung volumes, elastic recoil and compliance (Dyer 2012; Navaratnarajah & Jackson 2013). This decline in respiratory function leads to the collapse of smaller airways and air entrapment as expiration volumes are decreased (Dyer 2012; Feeney, Reynolds & Hussey 2011; Lalley 2013). Furthermore, diffusion capacity worsens with age, leading to a larger ventilation–perfusion mismatch, especially with exertion or exercise. Furthermore, an increase in work of breathing and a weaker cough effort causes a decrease in airway clearance (Dyer 2012; Lalley 2013; Sharma & Goodwin 2006).

Lung function and cough reflexes are generally impaired following abdominal surgery, irrespective of the patient’s age (Patman et al. 2017). The physical changes in the lung of an elderly person, therefore, could make the cough effort already less forceful and thus less effective (Dyer 2012; Lalley 2013; Sharma & Goodwin 2006), predisposing the elderly patient to diaphragmatic fatigue and respiratory failure when an extra load (such as abdominal surgery) is added (Sharma & Goodwin 2006). Elderly patients also show a poor response to hypoxia (low levels of oxygen) and hypercapnia (high levels of carbon dioxide), increasing their vulnerability to respiratory failure with surgery (Dyer 2012). The resulting shortness of breath, possibly worsened by comorbidities, may then lead to a decrease in quality of life (Dyer 2012).

The ageing process with subsequent lung alterations increases the risk of PPC in the elderly patient, but Karlsson et al. (2018) found that functional status (fit or frail) was a better predictor of PPC risk and postsurgical outcome than chronological age. Functional status can be defined as a person’s ability to perform physical and social activities necessary in daily routines and life roles (Van Cleave, Egleston & McCorkle 2011). Physical function declines with age, affecting both activity of daily life (ADL) and instrumental ADL (IADL) (Zasadzka et al. 2016). This decline can further be influenced by multiple factors such as frailty, comorbidities, strength and walking ability (Karlsson et al. 2018). These factors impact the patient’s ability to withstand surgery (Makary et al. 2010).

Almost half of elderly patients undergoing surgery already report lower functional levels (Hoogeboom et al. 2014). Additionally, hospitalisation and surgery further reduce the functional status of elderly patients through decreased activity, surgical stress, anaesthesia and complications (Petrucci et al. 2018; Pouwels et al. 2014). Petrucci et al. (2018) state that major surgery in elderly participants contributes to rapid loss of muscle strength, causing postural instabilities and negatively affects walking ability. This loss is aggravated by a decrease in activity following surgery. Studies show that elderly patients spend up to 80% of their recovery time in bed, severely compromising their independence (Hoogeboom et al. 2014; McComb et al. 2018).

Pre-operative studies focusing on both physical exercise and inspiratory muscle training have been shown to be effective in reducing PPC and preserving physical function in patients undergoing major abdominal (Barberan-Garcia et al. 2018; Dronkers et al. 2008; Katsura et al. 2015; Kulkarni et al. 2010; Mayo et al. 2011; Pouwels et al. 2014; Soares et al. 2013) and cardiac surgery (Katsura et al. 2015). These studies show that it is possible to improve physical function in patients in a short pre-operative timeframe (2–4 weeks of intervention) (Hoogeboom et al. 2014; Pouwels et al. 2014).

Pre-surgical exercise and education studies demonstrate that patients are better prepared physically as well as emotionally, thus improving outcomes (Hoogeboom et al. 2014). Concepts like ‘strong for surgery’ could significantly improve postsurgical recovery by decreasing complications such as PPC and improving general quality of life (Hoogeboom et al. 2014). It is currently not known whether a once-off education and exercise session with a home-based preparatory programme would have any effect in this population of interest. The purpose of our pilot study was to act as a starting point for investigating the effect of such a programme on clinical outcomes and physical function in elderly patients undergoing abdominal surgery when done once off in a South African context. Secondly, it aimed to highlight issues that could influence the feasibility of such a physiotherapy service.

## Method

Our study was a pilot single-blind randomised controlled clinical trial. Matched randomisation was done through a computerised random allocation generator by the second author. Participants were matched by Functional Comorbidity Index (FCI) score and age category. Matched randomisation allowed an even distribution of characteristics between the control and intervention groups. Group allocations were placed in sealed brown envelopes for concealment by the second author and given to the first author. The first author gave the sealed envelopes to a research assistant, for example, a physiotherapy practice receptionist, along with a chart to keep track of participants’ allocations. To ensure blinding, the first author did not have access to the envelopes or the chart during our study.

The study population consisted of elderly patients who were booked for elective abdominal surgery in a private hospital in Pretoria. Caring physicians (that is, general and gynaecological surgeons) referred potential study participants for inclusion. The participants were individuals 60 years and older, able to walk with or without a mobility aid prior to surgery, scheduled for elective abdominal surgery with at least 1 week prior to surgery and proficient in English. Patients were excluded from participation in the following instances: individuals with a known documented diagnosis of dementia or Alzheimer’s disease, individuals diagnosed with an acute respiratory tract infection within 2 weeks of potential study participation, the presence of cardiovascular instability in an individual (for example, unstable angina and New York Heart Association class 4 heart failure), the use of immunosuppressive medication for 30 days before surgery and previous surgery with associated chest wall manipulation.

The sample size was calculated using a power calculation. The threshold probability for rejecting the null hypothesis (alpha) was set at 5% and the probability of failing to reject the null hypothesis (beta) at 20%. Pilot studies are small studies that help design further larger studies that confirm the results (Arain et al. 2010). Our pilot study was calculated at 10% of a much larger project (Hertzog 2008). A previous study investigating the effect of pre-surgical intervention on functional capacity (by means of the 6MWT) after surgery reported an effect size of 7.1 m ± 11.5 m in the walking and breathing exercises group (Carli et al. 2010). When calculated, this resulted in an estimated sample size of 82 individuals for a larger study. To account for dropouts and missing data, this larger study aimed for a sample size of 100 participants. Thus, a minimum of 10 participants were required in our pilot study.

## Procedure

An education and exercise pamphlet was created by means of a narrative review of the literature with information extracted from the narrative review informing the pamphlet creation. Final content validation of the pamphlet was undertaken by a group of five expert peer-reviewers. The functions of the two physiotherapist research assistants were to conduct the once-off face-to-face pre-operative education and exercise sessions with the intervention group participants. Training of the research assistants ensured congruency between sessions. Following training, each research assistant treated the first author as they would a study participant. Minor adjustments and suggestions were made to further enhance similarity between research assistants when conducting face-to-face sessions with study participants. Data collection of the clinical study took place from November 2018 to December 2020. During the coronavirus disease 2019 (COVID-19) pandemic, all protocols were followed by all participants, research assistants and the first author.

Clinical outcomes measured included length of stay (LOS) in hospital, the development of PPC and the first time the patient mobilised out of bed. The development of signs and symptoms of PPC was identified by the Melbourne Group Scale (MGS) when reviewing participants’ clinical records. Functional outcome measures included the DeMorton Mobility Index (DEMMI), 6-minute walk test (6MWT), FCI and Lawton–Brody IADL scale.

The FCI was designed by Groll et al. (2005) to predict physical function rather than mortality based on a patient’s comorbidity data. The DEMMI, developed by De Morton, Davidson and Keating (2008), evaluates mobility, while the 6MWT measures functional exercise capacity (Crapo et al. 2002; Hijazi, Gondal & Aziz 2017). The Lawton–Brody IADL assesses the functional status of a patient (Vittengl et al. 2006), as the skills assessed rely on a combination of cognitive and physical function.

Physical function (DEMMI and 6MWT) was measured at three time points: baseline, hospital discharge and with doctor’s follow-up after hospital discharge. The other two parameters were measured twice; FCI at baseline and hospital discharge, IADL at baseline and follow-up after hospital discharge. Participants were evaluated by the first author following referral by the surgeon (baseline) a week prior to surgery. Once assessment was complete, a research assistant gave the control group a copy of the education pamphlet before leaving the research appointment.

In contrast, the intervention group participants received an education and exercise pamphlet together with a once-off face-to-face session with a physiotherapist, during which the entirety of the pamphlet was explained, demonstrated and questions answered. These exercises included bridging, back mobility exercises, resistance training and walking. Though exercises were generic, resistance bands were individually tailored according to the participants’ strength. Intervention group participants were requested to continue and diarise the exercises prescribed until surgery, and then resume with the exercises following surgery until follow-up. All participants were re-evaluated following surgery at the time of hospital discharge and again at their 2-week follow-up appointments with the relevant surgeon. During hospital stay, study participants (control and intervention) continued receiving physiotherapy care as per protocol in place at the clinical site. Treating physiotherapists were not made aware as to which group the participants were allocated.

## Data analysis

Data analysis was performed using IBM Statistical Package for the Social Sciences (SPSS) version 27 software. A statistician was consulted when needed. Intention to treat (ITT) analysis was done to account for missing data. Depending on the data, either the group average or the previous assessment’s data were imputed. The Shapiro–Wilk test was used to determine the normality of data. To determine differences between groups at baseline, the independent *t*-test, Pearson’s chi-squared test or Mann–Whitney *U*-test was used, dependent on whether data were normally distributed or not. Data were presented to two decimal points unless *p*-value was smaller than 0.01, in which case three decimal points were reported. Analysis over time looked at the change in the cohort as a whole (*n* = 12). A repeated-measures ANOVA test was used for this analysis.

### Ethical consideration

Ethical clearance was received from the University of Witwatersrand Human Research Ethics Committee (reference number: M180561). A clinical trial number was allocated from the Pan African Clinical Trial Registry (reference number: PACTR201809874713904). Permission and informed consent were obtained from all involved parties before commencing the study. These parties included relevant hospitals, physiotherapy practices, appropriate specialists and the participants.

## Results

**FIGURE 1:**
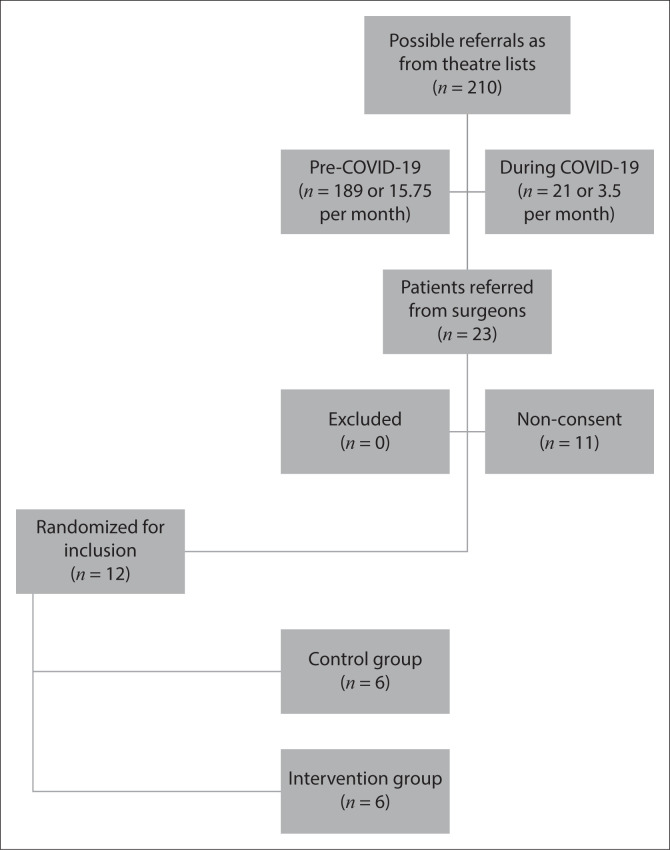
Number of participants and allocated groups.

The flow of study participants is outlined in Figure 1.

Our pilot study consisted of 12 participants (female, *n* = 11, 91.67%; male, *n* = 1, 8.33%), with a mean age of 65.75 (± 4.47) years and body mass index (BMI) of 27.89 (± 4.09) kg.m^2^. The demographics and physical function of study participants are presented in Table 1. The control and intervention groups were demographically similar at baseline except for BMI. It should be noted that the control group’s BMI is borderline normal compared to the overweight intervention group. Two control group participants did not live independently, as they had caregivers at home.

**TABLE 1:** Demographics and physical function of cohort at baseline (*n* = 12).

Demographic Mean (± SD), Median (IQR), % (*n*)	Cohort	*n* = 12	Control	*n* = 6	Intervention	*n* = 6	*p*	Effect size g	95% CI
Gender (% female, *n*)	91.67	11	83.33	5	100	6	0.36	−0.53	−1.59; 0.55
Age (years)	65.75	± 4.47	65.83	± 3.87	65.67	± 5.39	0.95	0.03	−1.01; 1.08
BMI (kg/m^2^)	27.89	±4.09	25.49	± 3.32	30.29	± 3.46	0.03	−1.31	−2.47; −0.09
Smoking history (% nonsmokers, *n*)	83.33	10	83.33	5	83.33	5	0.54	0.34	−0.73; 1.38
Activity levels (% active, *n*)	83.33	10	83.33	5	83.33	5	0.38	0.49	−0.59; 1.55
Living conditions (% independent, *n*)	75	9	66.67	4	83.33	5	0.55	0.33	−0.73; 1.38
Surgery type (% gynaecological, *n*)	83.33	10	66.67	4	100	6	0.20	0.78	−0.34; 1.86
Surgery method (% laparotomy, *n*)	83.33	10	83.33	5	83.33	5	1.00	0.00	−1.13; 1.13
FCI (conditions)	3	± 1.13	3.17	± 1.47	2.83	± 0.75	0.63	0.26	−0.79; 1.31
DEMMI (score out of 100)	79.50	74–96.25	87	67.50–100	79.50	74–85	0.74	0.19	−0.87; 1.23
6MWT distance (m)	435.50	364.75–458.50	448	293.50–468.25	435.50	393–441.25	0.67	−0.24	−1.28; 0.82
6MWT predicted† (m)	535.62	514.39–550.47	544.09	526.08–608.05	523.70	502.30–541.65	0.08	1.09	−0.08; 2.22
6MWT % of predicted distance (%)	81.90	64.63–87.62	79.82	52.40–88.66	81.90	73.69–86.11	0.47	−0.40	−1.45; 0.67
Lawton–Brody IADL (score out of 100)	100	100–100	100	85–100	100	100–100	0.36	−0.53	−1.59; 0.55

BMI, body mass index; DEMMI, DeMorton Mobility Index; kg/m^2^, kilograms per meter squared; FCI, functional comorbidity index; IADL, instrumetal activities of daily living; 6MWT, 6-minute walk test; SD, standard deviation; IQR, interquartile range; CI, confidence interval.

† , Equation from Duncan et al. (2015).

The FCI consists of 18 comorbidities, where severity is not specified. The following conditions were most prevalent among participants: arthritis and back conditions, respectively (*n* = 7, 58.33%; *n*_c_ = 4 and 3 respectively), visual impairments (*n* = 4, 33.33%; *n*_c_ = 3), osteoporosis (*n* = 3, 25%; *n*_c_ = 1) and upper gastrointestinal conditions (*n* = 3, 25%; *n*_c_ = 2). Conditions with only one response (*n* = 1, 8.33%) included chronic obstructive lung disease (control group), congenital heart failure (control group), stroke, diabetes (control group) and obesity. Both depression and angina were comorbidities listed by two (16.67%; *n*_c_ = 0 and 1, respectively) participants. When comparing the baseline percentage 6MWT distance achieved to the baseline FCI score, a strong negative correlation was observed (*r* = −0.792, *p* = 0.002). During testing with the 6MWT, the mean change in dyspnoea from pretest to post-test was 2.58 (± 2.23) and fatigue 1.83 (± 1.19) on the Borg Scale. Only two (16.67%) participants needed to stop and rest during testing, with one (8.33%) stopping short of the 6-minute mark. Participants mostly struggled with the balance component measured within the 15 hierarchical tasks of the DEMMI. Most participants (*n* = 11, 91.67%) were able to mobilise independently, with one (8.33%) requiring a walking stick. Table 2 provides information on the differences in clinical outcomes between the groups.

**TABLE 2:** Comparison of clinical parameters (*n* = 12).

Clinical parameter Mean (± SD) or Median (IQR) or % (*n*)	Cohort	*n* = 12	Control	*n* = 6	Intervention	*n* = 6	*p*	Effect size g	95% CI
LOS hospital (days)	4	3.25–4	4	2.50–4	4	3.50–4	0.59	−0.30	−1.34; 0.76
Surgery duration (minutes)	181.50	164–209.25	184	125.75–203.75	181.50	154.75–219	0.59	−0.30	−1.34; 0.76
PPC frequency (% yes, *n*)	50	6	66.7	4	33.3	2	0.57	0.60	−0.49; 1.66
Time from surgery to first mobilisation (hours, minutes)	20	± 2.44	20.21	± 2.16	19.41	± 2.83	0.59	0.29	−0.76; 1.34
Distance of first mobilisation (m)	130	85–225	90	60–245	177	100–242.50	0.86	−0.10	−1.14; 0.95

LOS, length of stay; PPC, postoperative pulmonary complications; SD, standard deviation; IQR, interquartile range; CI, confidence interval.

Clinical outcomes were not significantly different between the groups (Table 2). It is, however, important to note that fewer intervention group participants presented with signs or symptoms of PPC as assessed with the MGS, and they also walked further during their first time out of bed. Signs and symptoms of PPC included shortness of breath, productive cough with purulent sputum that influenced auscultation, oxygen saturation and chest x-ray findings. When applying the Pearson chi-squared test, it indicated that the baseline DEMMI score was significantly associated with the MGS (*p* = 0.03), but the 6MWT distance was not (*p* = 0.440). The higher the DEMMI score, the less likely the participant was to develop signs or symptoms of PPC as assessed with the MGS. Table 3 provides information related to the physical function parameters at hospital discharge.

**TABLE 3:** Comparison of physical parameters as measured at hospital discharge (*n* = 12).

Physical Parameter Mean (± SD) or Median (IQR)	Cohort	*n* = 12	Control	*n* = 6	Intervention	*n* = 6	*p*	Effect size g	95% CI
FCI (conditions)	2.67	± 1.03	3.17	± 1.53	3.67	± 1.86	0.28	−0.61	−1.68; 0.48
DEMMI (score out of 100)	74	74–82.25	74	69.75–76.75	74	69.75–85	0.71	−0.21	−1.25; 0.85
6MWT distance (m)	347	336.25–380	340.50	318.75–353	376	319.5–382.75	0.61	−0.28	−1.32; 0.78
6MWT % of predicted distance (%)	64.69	54.84–72.77	60.24	53–66.68	70.78	59.85–74.67	0.27	−0.63	−1.69; 0.47

6MWT, 6-minute walk test; DEMMI, DeMorton Mobility Index; FCI, Functional Comorbidity Index; SD, standard deviation; IQR, interquartile range; CI, confidence interval.

Control and intervention groups were comparable (*p* ≥ 0.05) at hospital discharge (Table 3). All study participants were discharged to their home from hospital. It is important to highlight that clinically, the intervention group was able to walk further during the 6MWT at hospital discharge and also reach a higher percentage of their predicted value of the 6MWT at this time point. Control and intervention groups were comparable (*p* ≥ 0.05) at follow-up after hospital discharge (Table 4).

**TABLE 4:** Comparison of physical parameters as measured with follow-up.

Physical parameter Median (IQR)	Cohort	*n* = 12	Control	*n* = 6	Intervention	*n* = 6	*p*	Effect size g	95% CI
DEMMI (score out of 100)	100	85–100	92.50	82.25–100	100	93.50–100	0.63	−0.26	−1.31; 0.79
6MWT distance (m)	413	369.75–439.25	422.50	368.25–440	397	333–426	0.84	0.47	−0.60; 1.52
6MWT % of expected distance (%)	74.60	63.73–83.24	74.27	62.65–83.35	74.60	63.45–83.32	0.15	0.1	−0.94; 1.15
Lawton–Brody IADL (score out of 100)	100	92.50–100	95	81.25–100	100	100–100	0.15	−0.90	−1.99; 0.24

6MWT, 6-minute walk test; DEMMI, DeMorton Mobility Index; IADL, Instrumental Activities of Daily Living; SD, standard deviation; IQR, interquartile range; CI, confidence interval.

Analysis within groups (cohort [*n* = 12], control [*n* = 6] and intervention [*n* = 6]) at different time points (baseline, hospital discharge and follow-up) was also done to determine the effect of the intervention on physical function as measured by the DEMMI and 6MWT, taking into account the time factor. Analysis over time looked at the change in the cohort as a whole (*n* = 12). A repeated-measure ANOVA determined that the overall DEMMI score differed statistically significantly between time points (*F*[2, 22] = 8.19, *p* = 0.002). Post hoc tests using the Bonferroni correction revealed that physical mobility, as measured by the DEMMI, decreases following surgery (81.08 (± 4.32) at baseline to 73.92 (± 2.67) at discharge); however, it was not statistically significant (*p* = 0.72). From discharge to follow-up (93.17 [± 3.07]), mobility again increased significantly (*p* = 0.002).

When analysing the control and intervention group separately to highlight between-group differences, the DEMMI score did not differ significantly between time points for the control group (*F*[2, 10] = 2.19, *p* = 0.16) but did differ statistically significantly for the intervention group (*F*[2, 10] = 8.96, *p* = 0.006). The Bonferroni correction showed that mobility decreased slightly from baseline to hospital discharge. Even though not statistically significant, it should be noted that the intervention group’s (79.50 [± 2.46] to 74.83 [± 4.19]) reduction in function was less than the control group’s (82.67 [± 8.67] to 73 [± 3.67]) reduction. The DEMMI scores improved following hospital discharge to follow-up after hospital discharge for both the control and intervention groups (90.67 [± 4.49], *p* = 0.10 and 95.67 [± 4.33], *p* = 0.05), respectively. The intervention group’s mobility improved significantly more than the baseline finding with follow-up assessment (*p* = 0.03) compared with the control group’s (*p* = 1.00).

A repeated-measure ANOVA with a Greenhouse–Geisser correction determined that the overall 6MWT (*n* = 12) score did not differ statistically significantly between time points (*F*[1.23, 13.51] = 4.24, *p* = 0.05). Further analysis, however, revealed statistically significant differences between control and intervention groups. The control group did not differ significantly between time points when analysed with the repeated-measure ANOVA and Greenhouse–Geisser correction (*F*[1.06, 5.29] = 1.64, *p* = 0.25). On the other hand, the intervention group conformed to sphericity and differed significantly (*F*[2, 10] = 18.18, *p* < 0.0005). Post hoc tests with the Bonferroni correction revealed that physical capacity, as measured with the 6MWT, decreased significantly following surgery (413.33 (± 22.85) at baseline to 350.17 (± 25.07) at discharge, *p* < 0.0005). From discharge to follow-up (376.33 [± 32.13]), mobility again increased, although not statistically significantly (*p* = 0.20) and not more than baseline.

### Challenges with recruitment

Recruitment rate was low, as only 10.95% of surgical patients were referred for possible participation. The number of elective surgical cases was also low because of the COVID-19 pandemic. A further 47.82% of referred patients did not give consent for participation. The explanations for nonconsent were not formally collected but included limited time following consultations, difficulty in travel arrangements and other commitments prior to surgery.

## Discussion

The elderly participants were reasonably healthy, with good mobility and fairly normal functional capacity, allowing them to participate independently with IADL. Based on existing evidence, participants were, however, considered at higher risk for complications following surgery because of their age. The risk, however, was lowered because of fair baseline physical function and laparoscopic lower abdominal surgeries (i.e. gynaecological surgeries) being performed rather than upper abdominal or open surgeries. Half of our pilot study participants developed signs and symptoms of PPC as assessed with the MGS, with twice as many cases presenting in the control group when compared with the intervention group. The incidence of PPC among high-risk patients undergoing abdominal surgery is reported to range from 16% to 20% (Boden et al. 2017; Mackay, Ellis & Johnston 2005). Boden et al. (2017) noted a significant difference in the incidence of PPC between their pre-operative physiotherapy group (12% incidence) compared to the control group (27% incidence; *p* < 0.001). Several studies report that prehabilitation is a protective factor against complications among high-risk patients undergoing elective abdominal surgery (Barberan-Garcia et al. 2018; Boden et al. 2017; Reeve & Boden 2016). Boden et al. (2017) report that prehabilitation reduced the risk of PPC by 52%, and Tahiri et al. (2016) report that participants who developed PPC took longer to return to baseline functional status.

The 6MWT has been shown to be effective in predicting the incidence of PPC in high-risk patients (Keeratichananont, Thanadetsuntorn & Keeratichananont 2016). Keeratichananont et al. (2016) found that a baseline 6MWT distance of 325 m or less predicted PPC with 77% sensitivity and 100% specificity. Flahive, Driscoll and Broderick (2018) also reported a statistically significant difference (*p* = 0.019) in the distance walked with the baseline 6MWT between participants who developed PPC (median 490 m [IQR 420–561.25]; age 71 years) and those who did not (570 m [502.5–630]; age 60 years). In contrast to these two studies, Paisani et al. (2012) found no difference (*p* = 0.57) in 6MWT distance between the participants who developed PPC (466 [± 97] m; 67 [± 12] years) and those who did not (485.3 (± 107.1) m; 57 (± 14) years). In line with Paisani et al. (2012), the results of our pilot study also indicate no association between baseline 6MWT distance and the development of signs or symptoms of PPC.

Our results indicate that the baseline DEMMI score significantly correlates with PPC findings (*p* = 0.03). To date, no studies report on the relationship between DEMMI score and PPC findings. Lawrence et al. (2004) did, however, report that better pre-operative function and the development of serious PPC regularly predict recovery. The data thus suggest that pre-operative physical function status may affect PPC, but further research is needed.

The distance participants mobilised with first uptime is very rarely reported; most studies only differentiate between less or more than 30 m. It should be noted that our study’s participants initially mobilised much further than 30 m (130 [85–225] m). Also of clinical note, if not statistically so, is that participants in the intervention group mobilised almost double the distance of those in the control group. The added distance to their first time up could be because of them realising the importance of ambulation post-operatively as an outcome of the education programme but potentially also because of engaging in more ambulation activity prior to surgery benefitting them postoperatively.

When participants were discharged from hospital, there were no significant differences between the intervention and control groups, as measured by the FCI (*p* = 0.28), DEMMI (*p* = 0.71) and 6MWT (*p* = 0.61). Dronkers et al. (2010) also report that the pre-operative programme did not significantly change the functional exercise capacity (6MWT) between groups. As seen in the results, intervention participants recovered past baseline in terms of DEMMI findings. The results, therefore, suggest that the intervention consisting of education and exercise with a physiotherapist may lead to improved mobility recovery following surgery. Said et al. (2012) report that following 2 weeks of hospital rehabilitation, DEMMI scores among 80-year-old patients with mobility issues improved with 9.6 (±8.8) points. This suggests that mobility improves with prehabilitaion as well as rehabilitation.

We found that the 6MWT distance decreased following surgery for both groups, as was expected. This reduction in 6MWT distance after surgery is supported by Carli et al. (2010). However, interestingly, the decrease was more significant in the intervention group. Both groups improved, but at posthospital follow-up, the intervention group had not yet returned to baseline; surprisingly, the control group improved past baseline. This suggests that other factors play an important role in the recovery of functional capacity. Antonescu et al. (2014) suggest that the minimal clinically important difference (MCID) of improvement as measured with the 6MWT distance over time for individuals who undergo abdominal surgery is 14 m (9–18). Both the control and intervention groups in our study demonstrated larger improvements in 6MWT distance walked in the postdischarge period than the suggested MCID of Antonescu et al. (2014). Minnella et al. (2017) suggest that prehabilitation enhances the recovery of functional capacity. Unfortunately, change in 6MWT distance during prehabilitation was not measured, but it would be interesting to know if pre-operative gains influenced the recovery of functional capacity.

A successful prehabilitation programme should involve the entire multidisciplinary team in order to optimise the short time frame from when the decision to operate is made to surgery. For the purposes of our study, surgeons were approached to refer possible participants, but they were not actively involved in our study. Study recruitment was hampered by low referral rates, and few individuals who were referred subsequently consented to participate. Feedback received why individuals declined participation indicated that they had limited time following surgeon consultations, difficulty in travel arrangements and other commitments prior to surgery. Karlsson et al. (2019), when evaluating the feasibility of a pre-operative supervised home-exercise programme in older adults undergoing colorectal surgery, found that reasons why individuals declined participation were that they had other time-consuming pre-operative examinations, no time for the intervention or were feeling stressed prior to surgery. Additionally, Northgraves et al. (2020) indicate that short surgical wait times prior to surgery influenced the feasibility of a prehabilitation exercise-based programme in individuals undergoing abdominal surgery. These findings related to recruitment challenges should therefore be considered as factors that may influence the feasibility of a pre-operative home-based education and exercise service to elderly individuals undergoing abdominal surgery.

Another limitation of our pilot study is that patients referred for participation were fit rather than frail. Kow (2019) suspected that frail patients have more to gain from prehabilitation than fit or previously frail patients. Two weeks of prehabilitation is the minimum time frame needed for patients to feel engaged and as if they worked to optimise their outcome (Shaughness, Howard & Englesbe 2018). Because of short theatre wait times, the time available for prehabilitation was limited to a maximum of 1 week. It would have been interesting to know which aspects of physical function had improved during prehabilitation and by how much. This was also not feasible as participants were only admitted to hospital the morning of surgery.

During the hospital admission, study participants continued receiving physiotherapy care as per protocol in place at the clinical site. Differences might have existed on how individual physiotherapists implemented this protocol during patient care, which could potentially have influenced patient recovery during hospital stay. This is an additional limitation of our study that should be taken into account when reviewing the findings. Future research is needed to explore the impact that protocol variability could have on patient outcomes.

## Conclusion

Our pilot study findings present some potential clinically significant changes when a once-off single pre-operative physiotherapy session with a home-based exercise programme is included into the surgical care regime of elderly patients undergoing abdominal surgery. Prehabilitation improved the uptake of early mobilisation when considering the distance mobilised by the intervention group during the first time up at hospital admission. Mobilising out of bed earlier and not experiencing PPC symptoms may have therefore led to a better 6MWT distance at hospital discharge. However, because of the small sample size, further research is necessary.

A strong negative relationship was noted in the FCI finding and 6MWT distance achieved at baseline. Surgeons could consider using the FCI to screen elderly patients during consultation prior to surgery. Frail patients, those with a suspected reduction in physical function because of increased FCI score, could then be referred for prehabilitation services. Van Cleave et al. (2011) suggest that three or more comorbidities indicate a marked decrease in function. Should patients not be able to participate in prehabiltation for any reason, an educational pamphlet would be more effective than no physical preparation for surgery.
