# Corrigendum: Pre-operative physiotherapy for elderly patients undergoing abdominal surgery

**DOI:** 10.4102/sajp.v79i1.1870

**Published:** 2023-06-23

**Authors:** Rozelle Labuschagne, Ronel Roos

**Affiliations:** 1 Department of Physiotherapy, Faculty of Health Sciences, University of the Witwatersrand, Johannesburg, South Africa; 2 Department of Physiotherapy, University of Pretoria, Pretoria, South Africa

In the published article, Labuschagne, R. & Roos, R., 2022, ‘Pre-operative physiotherapy for elderly patients undergoing abdominal surgery’, *South African Journal of Physiotherapy* 78(1), a1782. https://doi.org/10.4102/sajp.v78i1.1782, there was an error regarding the affiliations for Rozelle Labuschagne. As well as having affiliation 1, they should also have Department of Physiotherapy, University of Pretoria, Pretoria, South Africa.

The authors apologise for this error. The correction does not change the study’s findings of significance or overall interpretation of the study’s results or the scientific conclusions of the article in any way.

